# Identifying health related occupations of Twitter users through word embedding and deep neural networks

**DOI:** 10.1186/s12859-022-04933-2

**Published:** 2022-09-28

**Authors:** Kazi Zainab, Gautam Srivastava, Vijay Mago

**Affiliations:** 1 Department of Computer Science, Lakehead University, Oliver Road, Thunder Bay, ON Canada; 2 Dept of Math and Computer Science, Brandon University, 270 18th Street, R7A 6A9 Brandon, Canada; 3 Research Centre for Interneural Computing, China Medical University, No. 91, Xueshi Rd, North District, Taichung, 40402 Taiwan

**Keywords:** Deep learning, Natural language processing, Text classification, Medical data, Twitter

## Abstract

**Background:**

Twitter is a popular social networking site where short messages or “tweets” of users have been used extensively for research purposes. However, not much research has been done in mining the medical professions, such as detecting the occupations of users from their biographical contents. Mining such professions can be used to build efficient recommender systems for cost-effective targeted advertisements. Moreover, it is highly important to develop effective methods to identify the occupation of users since conventional classification methods rely on features developed by human intelligence. Although, the result may be favorable for the classification problem. However, it is still extremely challenging for traditional classifiers to predict the medical occupations accurately since it involves predicting multiple occupations. Hence this study emphasizes predicting the medical occupational class of users through their public biographical (“Bio”) content. We have conducted our analysis by annotating the bio content of Twitter users. In this paper, we propose a method of combining word embedding with state-of-art neural network models that include: Long Short Term Memory (LSTM), Bidirectional LSTM, Gated Recurrent Unit, Bidirectional Encoder Representations from Transformers, and A lite BERT. Moreover, we have also observed that by composing the word embedding with the neural network models there is no need to construct any particular attribute or feature. By using word embedding, the bio contents are formatted as dense vectors which are fed as input into the neural network models as a sequence of vectors.

**Result:**

Performance metrics that include accuracy, precision, recall, and F1-score have shown a significant difference between our method of combining word embedding with neural network models than with the traditional methods. The scores have proved that our proposed approach has outperformed the traditional machine learning techniques for detecting medical occupations among users. ALBERT has performed the best among the deep learning networks with an F1 score of 0.90.

**Conclusion:**

In this study, we have presented a novel method of detecting the occupations of Twitter users engaged in the medical domain by merging word embedding with state-of-art neural networks. The outcomes of our approach have demonstrated that our method can further advance the process of analyzing corpora of social media without going through the trouble of developing computationally expensive features.

## Background

### Introduction

Twitter is a popular social media platform providing micro-blogging service, in which users can share their views and opinions for up to 280 characters long messages known as “tweets” . Although, in other social networking sites such as LinkedIn and Facebook, users have the privilege of filling up their personal information in specific fields. Unlike these sites, on Twitter users can write a public outline about themselves in only 160 characters known as “Bio” . Not only tweets, but bios can also help to extract rich linguistic information, and can be extremely helpful for “user profiling”. User profiling is an active area of research since it helps to improve product recommendations and service quality. Besides, the prediction of user occupational class is highly vital for user profiling and for predicting users’ demographic features. In previous studies, multiple approaches have been proposed for predicting demographic attributes, which include composing multiple features that have been initiated from the text and network information of users for attaining best performances in terms of predicting classification tasks [1–6]. Users’ texts in social media have been studied extensively to mine the features hidden behind the textual data that include Spatio-temporal and social network information [7–9]. Earlier research has focused on examining users’ attributes such as gender, age, and location which showed that their attributes influenced their use of language [10–12]. The texts of the users enable us to analyze such properties [13–16]. For example, user profiling can be applied for designing recommender systems for cost-effective targeted advertisements [17]. In other words, it will be more useful to advertise the journals of the latest medical articles to a network of doctors and practicing physicians which are related to their medical fields than to users who might be working in IT sectors.

Based on the biographical content of the users on the Twitter platform, we aim to predict a user’s medical occupational class. Firstly, in our approach, a vector space model has been generated in which each vector represents the unique tokens of each biographical content. The vectors were later fed into Long Short Term Memory (LSTM), Bidirectional LSTM (BiLSTM), Gated Recurrent Unit (GRU), Bidirectional Encoder Representations from Transformers (BERT), and A lite BERT (ALBERT) neural network models respectively to perform multi-text classification. Our results have demonstrated that by combining neural network models with the Vector Space Model (VSM), our model has performed better in identifying the medical professions from the biographical contents. This task will be directly applicable in analyzing the medical professional trend on Twitter of users following Twitter medical accounts. We have focused on analyzing Twitter medical accounts since we can get a more concentrated network of users belonging to medical fields. Since users working in various types of medical field will be more interested in following such medical accounts than general users. This task can be applied in examining the users belonging to a wide range of medical fields. Health agencies can recruit users working in various medical fields for new job opportunities. For this study, we created a dataset of users following medical accounts, including their biographical content and a label belonging to an occupational class from the “National Occupational Classification”^1^ taxonomy. We have designed a dataset that is similar to the dataset that was built by Preoţiuc-Pietro et al. [3].

### Related Work

To predict the occupations of Twitter users a dataset was constructed by Preoţiuc-Pietro et al., assigning users in 9 hierarchical business job categories [3]. For predicting the user occupational class, word cluster distribution features from each of the users’ historical tweets were used. They were able to achieve 50% accuracy in their multi-classification task. Aletras and Chamberlain have built the user’s following connections from the users of the occupational dataset. The user embedding was used as an input into their classification model. They have deduced that major job categories may fluctuate consequentially with regions having inequalities of economic development [5]. Pan et al. have used users’ network information and their bio description, over their tweets for predicting their occupational group [6]. They have stated that bio description of users along with their network information, when used as features in the neural network graph, has improved the performance of predicting the occupational group compared to using the historical tweets and have attained an accuracy of 61% [18].

The task of identifying the occupation of users is a multi-classification problem as an individual has to be designated to one of many predefined categories which is a classic research area of machine learning [19]. Specifically for classification task, Deep Neural Networks (DNN) have demonstrated to perform better than conventional machine learning models [20–24]. One such enhanced model of DNN is the Recurrent Neural Network (RNN), which examines each of the words in the corpus and stores the semantics of past words inspected in hidden layers [25]. RNN has proved to be suitable for text classification problems as it can understand contextual information, which is highly required in learning the text semantically [26]. Many works have also proved that RNN based text classifiers perform better as it has achieved higher precision and accuracy scores. For instance, gated RNN was used for document-level sentiment classification [25] and sequential short-text classification based on RNNs [27]. However, RNN did not perform significantly well in memorizing from distant past texts, which means that RNN cannot remember the text that is at least five words behind the word that is currently being memorized by the model. To resolve this issue, the LSTM and GRU models based on RNN has shown to be very effective [28, 29]. A forget gate is added to the LSTM model which enables the model to forget the least important words. In contrast to the LSTM model, the GRU model has only two gates, a reset gate, and an update gate. The update gate plays a role that is similar to the forget gate. The gate decides which information is important to remember and adds it to its gate. The reset gate is responsible to decide how much of the past information should the model forget. The LSTM and GRU models are much more efficient since they can learn and remember more texts and solves the issue of memorizing longer texts [30]. LSTM and GRU models have been used extensively in the state of the art deep learning applications, like speech recognition, speech synthesis, and natural language understanding [31, 32]. Also, LSTM neural network was implemented in text classification tasks by combining LSTM and convolutional neural network [33]. Moreover, as the outputs of RNN are mostly based on the previous contexts, the Bi-LSTM model has been introduced [34]. This structure is effective in knowing the future contexts as it allows the networks to know both the backward and forward information about the sequence at each time step. Bidirectional-LSTM runs inputs in two ways, one from the previous texts to upcoming texts. Similarly, one from upcoming contexts to previous contexts. In contrast, to this approach, LSTM is uni-directional and Bi-LSTM has shown to outperform LSTM as Bi-LSTM at any point in time can preserve information from not only the previous contexts but also from the future contexts. BERT is also a bidirectional model just like the Bi-LSTM model and its major component is that it uses a bidirectional transformer language model while training on textual data [35]. A Transformer is a machine learning model that considers the ordered sequence of the data^2^. BERT uses two pre-training techniques [35].

- Masked language model- Masking is carried out in three different ways. For example, if the sentence to be trained is “My dog is hairy” [35] and the word “hairy” is selected to be the token, then masking is done either by replacing it with a $<Mask>$ token i.e., “My dog is $<Mask>$” or with a random token e.g. “My dog is an apple” or by keeping it as it is i.e., “My dog is hairy” [35]. To get the context of a word these three methods are used together [36].
- Next Sentence Prediction - The model is given a pair of sentences and the model has to predict whether the second sentence comes after the first sentence or not in the corpus [35, 37].

This is the reason why BERT is significantly different from the other textual classifiers [35].

As the quantity of training data and the model size increases, the performance of the model increases [38]. However, with the increase in model size, it becomes difficult to pre-train the model because of the Graphical Processing Units (GPU) memory limitations and it takes longer training times [38]. ALBERT was introduced to solve this issue. ALBERT has the same architecture as BERT. However, ALBERT uses two parameter-reduction techniques to significantly decrease the number of training parameters of BERT. They are:

- Factorized embedding parameterization [38], splits down the larger word matrix into smaller matrices so that the complexity is reduced [36].
- Cross-layer parameter sharing stops the parameters from increasing as the depth of the neural network increases [38].

Both the techniques drastically reduce the training time of the model [38]. With the growing importance placed on collecting data, it has now become possible to develop more efficient and trustworthy classification methods to study and analyze biographical data for user profiling tasks. Our contributions are summarized as follows:

1. We propose a method developed on word embeddings
2. We amalgamate the word embeddings with neural network models to predict the medical occupations
3. We analyze real-world Twitter data of users users from their biographical contents
4. We assist in the design process of more effective recommender systems.

## Results

The experimental evaluation of the architecture has been performed on a computer with a 64-bit core i7 processor running Windows 10 and 64 GB of RAM. The CPU host is a 128-bit quad-core Intel Xeon E5520 with a clock speed of 3.27 GHz. The GPU device is an NVIDIA Tesla C2075 with 448 CUDA cores (16 multiprocessors with 32 cores each) and a clock speed of 1.15 GHz. It has 2.8 GB of global memory, 49.15 KB of shared memory, and a warp size of 32. Both the CPU and GPU are used in single precision. All code and experimental analysis details are available through request from our laboratory website^3^

We have calculated the recall, accuracy, and precision weighted scores to evaluate the performance of our approach of combining word embedding and each of the models respectively. As our dataset is highly imbalanced, we have used weighted metric scores as shown in Table 1. As the weighted scores consider the label imbalance issue into account. We have used the scikit-learn library for computing the metric scores [39]. The results have shown that ALBERT has performed significantly better compared to the other neural network models. ALBERT has achieved the highest accuracy, precision, recall, and F1-score. Furthermore, we have compared the performance of these models with the traditional machine learning models as well, to validate whether the DNN models perform better than the traditional machine learning models. Overall from the scores, we can see that the DNN model outperformed in predicting the occupational classes of the users. This may be because DNNs try learning high-level features from the textual data. Moreover, due to the availability of high-end, robust computational engines like GPU, it is possible to execute the DNNs. Whereas, for machine learning algorithms, domain expertise is highly required for hardcore feature extraction which makes the task much more challenging. Among the DNN models, ALBERT, BERT, Bi-LSTM, and LSTM have higher precision scores compare to recall scores which suggests that these models predicted more true positives than false positives. Besides, the GRU model has a higher recall value compare to the precision score which shows that GRU can correctly identify more true positives and fewer false negatives. ALBERT took the least amount of time for being trained with the corpus due to its parameter-reduction techniques. In contrast to ALBERT, BERT took the highest amount of time for training. Moreover, as ALBERT takes fewer parameters compared to the BERT model, the training period of ALBERT is less than the amount it took to train BERT. BERT models are already pre-trained with Wikipedia (2.5B words) and BookCorpus dataset (800M words) [38]. As the BERT models are already pre-trained and it is capable of learning complex features faster than the other DNNs.

**Table 1:** Metrics scores

Model	Accuracy	Precision weighted	Recall weighted	F1 weighted score	Training time (s)
Naïve-Bayes	0.73	0.62	0.60	0.64	586
LR	0.78	0.70	0.70	0.70	574
SGD	0.79	0.74	0.78	0.75	607
GRU	0.89	0.83	0.86	0.88	357
LSTM	0.90	0.89	0.85	0.84	453
Bi-LSTM	0.91	0.86	0.85	0.88	762
BERT	0.94	0.90	0.89	0.89	908
ALBERT	0.95	0.92	0.91	0.90	350

We have also generated a heat map to analyze the performance of all the classes in each of the models which are shown in Fig. 5. The color of the heat map changes from red to blue, from 0.0 to 1.0 respectively where 0.0 represents the lowest score and 1.0 represents the highest score. From Fig. 5d and h we can see that BERT and ALBERT were able to identify and classify most of the classes compare to the other models with the metric scores ranging from 0.6 to 1.0. Although there were fewer data points for classes 6 and 7, yet it could predict the classes better than the rest of the models. The number of data points for each of the classes is shown in Table 3. The Bi-LSTM model was able to classify the classes which have higher data points compared to the classes which have fewer data points shown in Fig. 5b. It predicted classes 1, 2, and 5 which has a better score for precision, recall, and F1 score. Followed by the LSTM model in Fig. 5a which could predict classes 1,2 5 properly compare to classes 3 and 4 which has fewer data points than the classes 1, 2, and 5 as the intensity of blue color fades in the regions of classes 3 and 4. Similarly, the GRU model in Fig. 5c, the metric scores shows that the model also performed well for classes 1, 2, and 5 but it performed lower than the LSTM model when predicting the classes of 3 and 4. However, GRU performed slightly better than LSTM and Bi-LSTM in predicting the classes of 6 and 7 which have the least data points. Out of the machine learning models, Naïve-Bayes in Fig. 5e performed the worst as it could neither predict the classes which had higher data points and lower data points properly. As, for classes: 1,2,3,4,6, and 7 the score ranges from 0.0 to only 0.5. Compare to Naïve-Bayes model, LR and Stochastic gradient descent (SGD) in Fig. 5f and g have performed better. Overall, most of the models were able to predict classes, which have higher data points compared to the classes that have lower data points. This suggests that with an increasing number of biographic contents for each of the classes, the performance of the models would increase especially of the Neural Network models. One of the reasons why most of the models performed with a lower score for classes 6 and 7 is because the job titles vary a lot in these 2 classes as it has several minor groups, and many of the job titles belong to these categories are not that much common.

Based on our observations, we can conclude that the neural network models especially ALBERT perform well as the neural network classifier along with word-embedding may have extracted linguistically rich semantic information embedded in each biographic content to the ALBERT classifier and so this approach results in better classification performance.

## Conclusion

In our approach, we have combined word embedding with deep learning neural network models for predicting the professions of users working in the medical fields. Our research has shown that by composing word embedding with neural network models it has outperformed the conventional machine learning classifiers in identifying the medical occupations of users. Moreover, traditional classification methods depend mostly on features developed by human intelligence which can be very challenging to design. Hence, text classification tasks can tend to become very slow when there is a burden to construct any particular attribute or feature. Thus, classification tasks can be executed much faster when there is no need to design any feature or specific attribute. In the future, the medical occupation dataset can be further extended by adding the network features from each of the users. Network features can be pre-processed from the follower/ following IDs from each of the users, and the features generated from the social network graphs from each of the users can further contribute to improve the occupation prediction of the users. The main contributions of this research involved the Natural Language Processsing methodology used on Twitter data, the deep Neural Networks that were used are standard tuned specifically to use with the word embedding frameworks. In the future, more specific Neural Networks should be used accompanied by an in-depth ablation study.

## Methods

In this paper, our task is a multi-class text classification problem where we want to identify the most likely medical profession for a given user based on their biographical content. At first, we have generated a vector space model in which each vector represents the unique tokens of each biographical content. Then the vectors are fed into LSTM, Bi-LSTM, GRU, and BERT neural network models respectively. These models then perform multi-classification, identifying the occupation of the user. Our results have shown that neural network models along with Vector Space Model (VSM) have performed well in identifying the medical professions from the biographical contents.

### Our approach

At first, from the unlabeled raw data, a term index Vector Space Model (VSM) is generated, in which the vectors depict the position of the unique tokens in the textual data. Then, a sequence of vectors representing each bio content is produced using the VSM and given as input into an LSTM, Bi-LSTM, GRU, and BERT neural network models respectively that executes a multi-classification task. We, at first, converted our occupation classification into a sequence classification problem, so that the task can be managed appropriately by the neural network classifiers. An example of a biographical content identified of a user belonging to the pharmacist category can be seen in the text ’Pharmacist, professor and specialist in geriatrics’, where the pharmacist category can be extracted accordingly.

### Representation of the biographical data

Our system specializes in feeding raw corpus into the deep learning classifiers. One of the major drawbacks of textual data is, these data cannot be directly represented as dense vectors as the terms from each text tend to have variable lengths. To solve this issue, distributed representations of words (word embedding) are used, where the terms in the text are formatted as dense vectors. This can be done with the aid of padding (“pad”). Padding allows us to get a fixed size input for the vocabulary of all the unique tokens in the corpus. In the vocabulary, the texts cannot be directly portrayed as vectors, and to resolve this issue, the unique tokens are placed with their indices. The biographical texts are converted into a sequence of indices (positive integers), instead of a sequence of tokens. Then, a dense vector is created from the term index sequences, where their representations are aligned to that of the original text. Previous works have also shown that the distributed representation of words in vector space is embedded with rich semantic and syntactic information [40–42]. Therefore, we created a vector space model from the term index representation of the texts in which the dense vectors were built from the biographical contents’ index-terms. By representing the data in this approach, we expect to feed the classifiers with the crucial knowledge embedded in each of the textual contents.

**Table 2:** Text, Tokenized term indexes

Text	Pharmacist	professor	and	specialist	in	geriatrics
Tokenized term index	[ $i_{Pharmacist}$ ]	[ $i_{professor}$ ]	[ $i_{and}$ ]	[ $i_{specialist}$]	[ $i_{in}$ ]	[ $i_{geriatrics}$ ]
Original term index	467	43	254	78	51	165

In Table 2, the first row shows the sequence of tokens (or term) of the biographic content. The tokenized representation of indices *i*(term) in the vocabulary of each of the terms is shown in the second row. Lastly, the third row shows the order of the original term indices where the values are determined by the locations of the terms in the vocabulary.

**Fig. 1:**
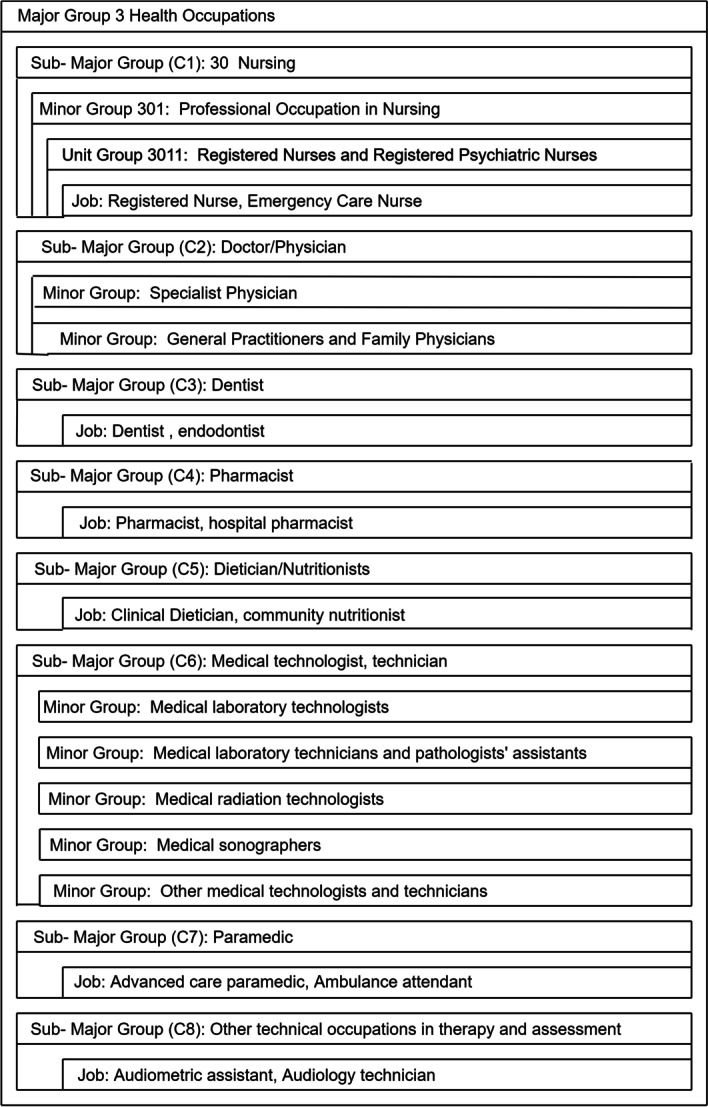
Subset of the NOC classification hierarchy

### Twitter biographical data of users

As far as we are aware, there are no existing datasets available that provide a convenient way to predict the medical professions of users. Hence, we have built a dataset that maps users to their medical occupations based on their biographical content. Similar to the approach taken by Preoţiuc-Pietro et al., we have examined the Twitter users following Twitter medical accounts having more than 100K followers. The Twitter users who had mentioned their medical profession in the Twitter user description field (“Bio”), those description fields were annotated. The users were selected from 1% of the sample taken through the Twitter API [43]. By analyzing the description fields of 45,000 users approximately, we found out the following categories: empty description (10.75%), random contents (17.23%), found user information but not related to medical occupations (55.87%), and medical occupations related information (16.15%).

To map the Twitter users to their respective occupations, we have used the standardized job classification taxonomy which is provided by the National Occupational Classification (NOC). NOC is a Canadian government system, which is developed by the Office of National Statistics for categorizing occupations. The NOC scheme consists of seven major medical professional groups coded with digits from 1 to 7. Each major group is split into sub-major groups that are enlisted with 2 digits, in which the first digit represents the major group and the second group represents the sub-major group. Each sub-major group is further categorized into minor groups coded with 3 digits. The minor groups are further broken down into unit groups, which are indicated with 4 digits. The jobs are classified hierarchically based on the skill requirements which are suitable for each of the jobs. The unit groups are the end-leaves of the hierarchy and highlight the appropriate jobs related to each of the major groups. Figure 1 shows a part of the NOC hierarchy. Even though there are several other existing hierarchies, we base our research on the NOC classification list since it has been published recently in 2016 and each of the major groups has also added the latest jobs and provided a larger range of job titles, which was highly important in generating our dataset.

For building the dataset, 4-digit NOC unit groups were used to find suitable medical job titles that suited best with the users’ medical occupation-related bio contents. As the NOC unit groups have precise medical job designations. The user accounts were combined into minor (3 digits) categories. To improve the quality of the dataset, the accounts with their bio descriptions were manually examined and the accounts of organizations and companies were removed which had no descriptions related to minor category medical occupations. Moreover, to ensure that the users were not having any fake accounts, 3-digit minor categories users’ accounts that had less than 50 user accounts in their follower list were removed. Finally, a total of 6754 users from the minor categories were combined in 7 sub-major NOC categories. The seven groups were distributed as such: 25%, 31.6%, 3.8%, 9.1%, 27.1%, 1.7%,1.33%, 0.31% (according to the classes in Fig. 1) and the number of users for each of the classes are shown in Table 3.

**Table 3:** The table shows the number of classes (left column) and classified jobs with multiple sub-major groups (middle column) by National Occupation Classification

Occupational class	National occupation classification	Users
1	Nursing Occ.	1691
2	Doctor/Physician Occ.	2137
3	Dentist Occ.	257
4	Pharmacist Occ.	614
5	Dietitian/Nutritionist Occ.	1831
6	Medical technologist, technician Occ.	112
7	Paramedic Occ.	90

The right-most column represents the number of users

To build the vocabulary and VSMs, the preprocessed corpora of unlabeled biographic contents were used for the biographic contents of each of the users. A guideline of annotations was developed to annotate the biographic contents properly. The guideline explains how to identify the occupation of the individual users, and includes descriptions and examples of the medical professions. The bio contents were divided into four portions and four annotators annotated each set of texts separately. Then, the labeled texts were further reviewed by expert annotators. The dataset was divided into the ratio of 70:30 for training and testing size.

### Data processing

**Fig. 2:**
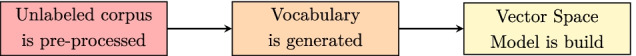
The generated vocabulary and VSM from the non-annotated texts

**Fig. 3:**
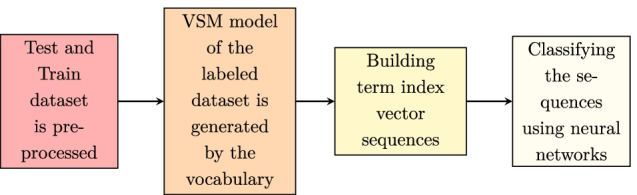
Shows the input sequence of vectors into the neural networks for classification. (Figure 4 describes the architecture in detail)

The vocabulary and VSM were created from the non-annotated biographical contents and represented these texts as a sequence of dense vectors. Then, we classified the contents with LSTM, Bi-LSTM, GRU, BERT, ALBERT neural networks respectively. At first, a corpus of 6754 unlabeled medical occupation-related bio contents was preprocessed in which URL links and emojis were filtered out. Certain punctuation, duplicates, and texts with URLs were also removed. Secondly, each unique individual index terms were combined to create a vocabulary, and finally, a VSM was generated from the preprocessed textual terms. Figure 2 below shows the step by step process of how all unique terms in the vocabulary was generated and how the VSM model was constructed from the vocabulary. The process of representing each biographical content of the annotated corpus into a sequence of index vector is shown in Fig. 3. In this process, the model at first searches in the vocabulary for the locations of the biographical content terms and then tracks down the required dense vectors from the location information. Finally, the vectors are arranged accordingly for generating a sequence of term index vectors.

**Fig. 4:**
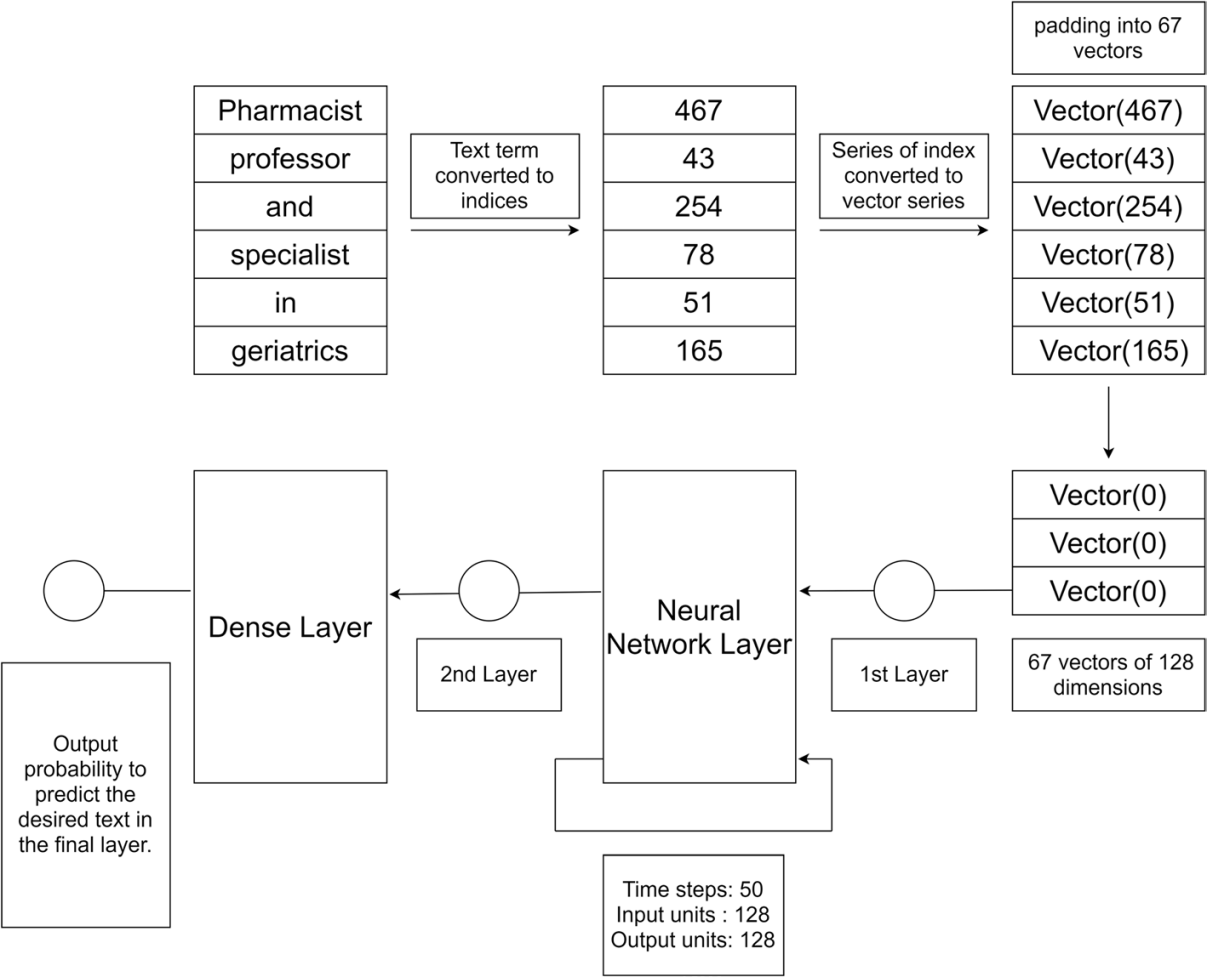
Architecture of the Model

**Fig. 5:**
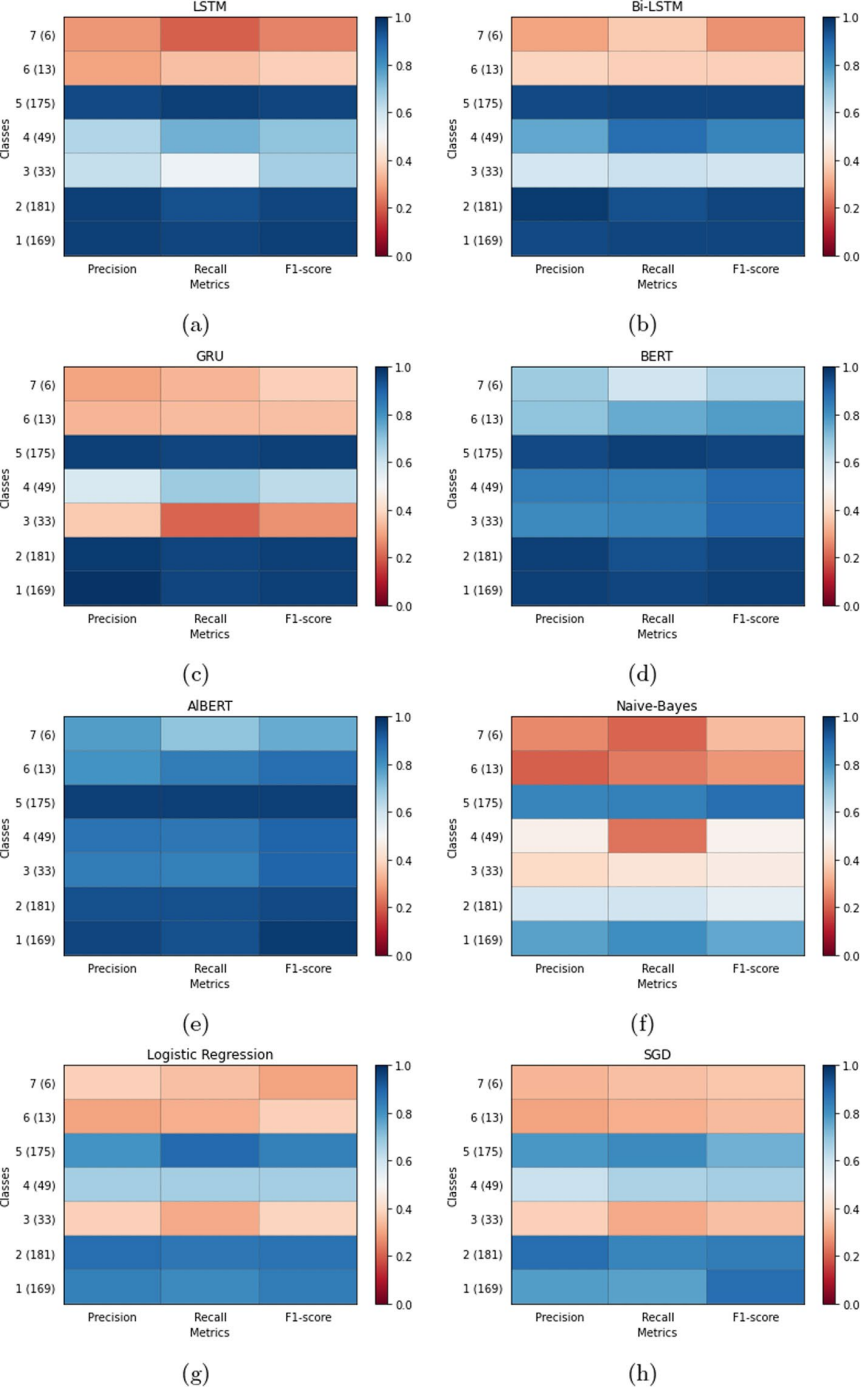
Precision, Recall, F1-Score Metrics of various models. Sub-figure (**a**) highlights the LSTM model, (**b**) Bi-LSTM model, (**c**) GRU model, (**d**) BERT model, (**e**) AlBERT model, (**f**) Naive-Bayes model, (**g**) Logistic Regression model, and (**h**) SCD model

### Implementation

The architecture of the neural network model for this study is shown in Fig. 4. The text of each biographical content is compiled as a sequence of 67 index term vectors as 67 was the largest number of terms collected from each of the contents. If in case, a text was less than 67 index terms, then the sequence was appended with the padding of indices. Each index term was formatted as a 128-dimensional vector of the index corresponding to the term in each of the biographical content. For each text, a sequence of 67 vectors of 128 dimensions was fed to each of the neural network classifiers. The neural network models executed the input sequence and generated the output. Our neural network models were based upon the implementation in Keras, and TensorFlow was used as the backend and high-end Graphics Processing Units (GPU) were used to execute the DNNs. Each of the models was composed of three layers: word embedding layer, LSTM/BiLSTM/GRU/BERT/ALBERT layer, and the final dense layer to process the results. Each of the models was trained individually, and accuracy was recorded in each of the epochs. We have observed that the accuracy became stable at around 15 epochs, on average for all the models; so we have selected 15 epochs for training our models (Fig. 5).

## Data Availability

All raw data, training sets, testing sets, and code used during the experimental analysis is available(https://github.com/ZainabKazi22/occupation_twitter).

## Abbreviations

LSTM: Long short term memory
GRU: Gated recurrent unit
BERT: Bidirectional encoder representations from transformers
ALBERT: A lite bidirectional encoder representations from transformers
NOC: National occupational classification
VSM: Vector space model
DNN: Deep neural network
GPU: Graphics processing units
SGD: Stochastic dradient descent

## Footnotes

1 https://noc.esdc.gc.ca/

2 https://www.researchgate.net/publication/344158120_A_Survey_on_Language_Models

3 https://www.datalab.science/
